# Daytime mid-latitude F_2_-layer Q-disturbances: A formation mechanism

**DOI:** 10.1038/s41598-020-66134-2

**Published:** 2020-06-19

**Authors:** Loredana Perrone, Andrey V. Mikhailov, Anatoly A. Nusinov

**Affiliations:** 1 Istituto Nazionale di Geofisica e Vulcanologia (INGV), Rome, Italy; 2 Pushkov Institute of Terrestrial Magnetism, Ionosphere and Radio Wave Propagation (IZMIRAN), Moscow, Russia; 3 Fedorov Institute of Applied Geophysics (IAG), Moscow, Russia

**Keywords:** Space physics, Astronomy and planetary science

## Abstract

Negative and positive near noontime prolonged (≥3 hours) F_2_-layer Q-disturbances with deviations in N_m_F_2_ > 35% occurred at Rome have been analyzed using aeronomic parameters inferred from f_p180_ (plasma frequency at 180 km height) and f_o_F_2_ observations. Both types of N_m_F_2_ perturbations occur under quiet (daily Ap < 15 nT) geomagnetic conditions. Day-to-day atomic oxygen [O] variations at F_2_-region heights specify the type (positive or negative) of Q-disturbance. The [O] concentration is larger on positive and is less on negative Q-disturbance days compared to reference days. This difference takes place not only on average but for all individual Q-disturbances in question. An additional contribution to Q-disturbances formation is provided by solar EUV day-to-day variations. Negative Q-disturbance days are characterized by lower h_m_F_2_ while positive – by larger h_m_F_2_ compared to reference days. This is due to larger average Tex and vertical plasma drift W on positive Q-disturbance days, the inverse situation takes place for negative Q-disturbance days. Day-to-day changes in global thermospheric circulation may be considered as a plausible mechanism. The analyzed type of F_2_-layer Q-disturbances can be explained in the framework of contemporary understanding of the thermosphere-ionosphere interaction based on solar and geomagnetic activity as the main drivers.

## Introduction

Usually F_2_-layer disturbances are related to geomagnetic activity variations but there is a class of F_2_-layer perturbations which occur under quiet geomagnetic conditions (Q-disturbances), their magnitude being comparable to moderate F_2_-layer storm effects. Day-to-day N_m_F_2_ variability is 15–20% at middle latitudes^1,2^ while we are speaking about N_m_F_2_ deviations with the magnitude >35% occurring near noontime with a duration more than three hours, i.e. longer than two characteristic e-fold times of the daytime F_2_-layer (the e-fold time is the time required for a parameter to change by 2.72 times). Such long-lasting N_m_F_2_ deviations imply either changes in the ionizing solar EUV radiation or changes in the production/recombination rate (i.e. thermospheric neutral composition) or/and changes in vertical plasma drift mainly related to thermospheric winds. Variations of electric field cannot be excluded but this is less probable during magnetically quiet periods analyzed in the paper.

There is a widely spread opinion that F_2_-layer Q-disturbances are related to the impact from below – the so-called “meteorological control” of the Earth’s ionosphere^1–6^. However, one has to be cautious to associate all quiet-time F-region disturbances with forcing from the lower atmosphere. Nevertheless accepting that geomagnetic activity is a major cause of F_2_-layer day-to-day variability Rishbeth^2^ stressed that the ‘meteorological’ impact at least was comparable to the geomagnetic one. Despite lots of publications on this topic the actual mechanism of observed day-to-day N_m_F_2_ variations has not been revealed yet. One may find^7,8^ that this variability is attributed to ‘solar’, ‘geomagnetic’ and ‘other’ causes. Therefore any attempt to give a quantitative explanation to day-to-day N_m_F_2_ changes should be considered as one more step towards understanding of the F_2_-layer variability and its prediction, the latter being very important from practical point of view.

The morphology and possible mechanisms of Q-disturbances were earlier discussed^9–12^. It was shown that both negative and positive daytime Q-disturbances may be related to thermospheric circulation and neutral composition changes. However that time we had not either necessary aeronomic parameters or solar EUV observations to explain the observed Q-disturbances and the number of Millstone Hill ISR observations of Q-disturbances used in previous analyses was very limited. Now using the recently developed method^13^ to extract aeronomic parameters from ground-based ionosonde observations, we can reanalyze and specify the formation mechanisms of positive and negative Q-disturbances at least at middle latitudes for daytime hours. Moreover today we have direct solar EUV observations and satellite neutral gas density measurements which can be successfully used to control the obtained results.

The proposed method^13^ can be applied when bottom-side Ne(h) profiles are available and such possibility now exists with the worldwide DPS-4 digisonde network^14^. However for various reasons the accuracy of Ne(h) profiles at F_1_-layer heights (used in the method) is different at different stations and not all of them can be used for our aims. There are two ionosondes at Rome (41.9°N; 12.5°E) and DPS-4 observations can be controlled by Italian ionosonde (http://www.eswua.ingv.it/ingv/home.php?res=1024). For this reason Rome DPS-4 Ne(h) observations were used for physical interpretation.

The aims of the paper may be formulated as follows.

- to find available cases of negative and positive daytime Q-disturbances using Rome ionosonde observations;
- to reveal aeronomic parameters responsible for the formation of daytime Q-disturbances in question;
- to give physical interpretation to the observed daytime Q-disturbances, i.e. to specify their origin.

## Method

Selection of the background level is a crucial point dealing with Q-disturbances and various approaches are used to specify it. Our earlier method^9^ was based on 27-day running median centered to the day in question. Monthly median or running medians calculated over previous ∼ 30 days are often used as the background^15–17^. However any median includes the effects of geomagnetic disturbances occurred during the analyzed period therefore different periods turn out to be in different conditions. Better results should give a selection of magnetically quiet days at a station with binning them in terms of hour, month and range of solar activity. The mean value for each bin provides a quiet-time background level which can be applied with suitable interpolation to any day of a month^18–21^. But Q-disturbances inevitably contribute to such background level.

Another direction is based on using model monthly median f_o_F_2_ as the background level^22,23^. After averaging (in the model) of many monthly medians obtained under various geomagnetic conditions but similar levels of solar activity one may hope that such average median presents a background level corresponding to a given level of solar activity. Both positive and negative F_2_-layer disturbances (including Q-disturbances) should be seen with respect to such background level.

We may also derive local (for each station) monthly median f_o_F_2_ models^24^ which are based on the ionospheric T-index^25,26^ as an indicator of solar activity level. It is well-known that effective ionospheric indices of solar activity provide the best correlation with monthly median f_o_F_2_ ^27^. Such model medians derived for each station are used as the background to calculate f_o_F_2_ deviations.

Q-disturbances in our analysis are referred to average over 11, 12, 13 LT hourly (N_m_F_2_/N_m_F_2med_ − 1) × 100% > 35% if all 3-hour *ap* indices were ≤ the threshold for 24 previous hours. The analyzed negative Q-disturbances took place under very low geomagnetic activity – the average threshold over 24 previous hours on *ap* was 6.1 ± 2.53 while the average threshold for positive disturbances was slightly higher – *ap* = 10.1 ± 3.87. A 24-hour preceding time interval was chosen basing on the empirical estimation of the ionosphere reaction to forcing geomagnetic activity. Some estimates of this time constant for mid-latitude F_2_-region are: 12 h^18^, 15 h^28^, 6–12 h^7^; 16–18 h^16^, 8–20 h depending on season^29^.

The analyzed type of Q-disturbances are not numerous, the larger their magnitude the less they are in number. Our previous morphological analysis^9^ has shown that positive Q-disturbances are much numerous compared to negative ones and this is valid especially for noontime perturbations. The selected disturbances should be strong enough to be outside of usual day-to-day 15–20% N_m_F_2_ variability. Further, only the period since 2002 can be analyzed as daily EUV and satellite neutral gas density observations used for a comparison are available for this period. All this taken into account together has specified the magnitude of selected disturbances δN_m_F_2_ = N_m_F_2 Qday_/N_m_F_2 ref_ > 35%. Therefore negative disturbances listed in Table 1are all corresponding to the listed requirements which could be found for the analyzed (2002–2018) period.

**Table 1:** Negative and positive F_2_-layer Q-disturbances at Rome along with daily F_10.7_, 0.5(F_10.7_ + F_10.7 81_), and *ap(τ)* indices (at 12 LT) for Q-disturbance and reference days (second line). N_m_F_2_ (in 10^5^ cm^−3^) are averaged over the observed at (11-13) LT values.

Negative Q-disturbances					Positive Q-disturbances				
Date	F_10.7_	(F + F_81_)/2	ap(τ)	N_m_F_2_	Date	F_10.7_	(F + F_81_)/2	ap(τ)	N_m_F_2_
13/06/2002 08/06/2002	133.4 155.2	147.3 157.4	6.1 5.1	6.19 10.5	23/08/2002 08/08/2002	224.5 134.6	203.8 57.6	6.1 3.9	15.8 8.54
17/10/2004 23/10/2004	91.9 131.6	99.9 119.4	2.3 2.3	6.61 9.17	17/06/2004 28/06/2004	111.3 89.4	109.397.5	7.2 9.2	8.14 5.73
15/08/2005 28/08/2005	75.8 89.8	83.9 90.1	4.2 4.1	2.94 4.97	26/10/2004 23/10/2004	136.7 131.6	121.6119.4	4.2 2.3	14.2 9.17
14/02/2006 19/02/2006	77.3 76.5	77.7 77.3	1.0 5.1	4.08 5.85	10/06/2007 06/06/2007	75.9 84.6	74.979.6	8.1 2.1	5.57 3.85
27/01/2010 11/01/2010	77.8 89.2	79.8 84.9	1.8 6.0	3.02 3.98	22/10/2009 13/10/2009	71.6 69.8	72.170.9	8.9 2.5	6.61 5.40
06/02/2010 11/02/2010	87.5 94.2	85.0 88.6	1.6 5.0	3.84 5.24	15/02/2010 11/02/2010	87.6 92.4	85.588.6	8.2 5.0	8.96 5.24
18/12/2011 05/12/2011	127.4 158.1	134.6 150.8	1.1 1.3	6.97 12.4	20/10/2010 03/10/2010	83.9 80.0	83.180.4	4.7 1.7	8.75 5.90
14/01/2012 07/01/2012	132.3 140.5	129.9 136.1	3.2 6.4	6.19 8.82	11/06/2012 27/06/2012	133.9 106.3	130.8116.9	7.2 4.4	10.7 6.08
06/12/2012 15/12/2012	97.4 122.4	109.1 122.1	1.2 6.2	5.79 8.47	30/09/2012 12/09/2012	135.6 102.6	127.2111.3	6.6 4.2	17.0 8.54
07/12/2012 15/12/2012	97.1 122.4	109.1 122.1	0.2 6.2	5.13 8.47	25/08/2013 01/08/2013	112.6 112.1	111.0112.7	5.7 4.1	10.5 6.61
17/04/2013 04/04/2013	106.3 128.5	115.3 124.5	3.1 4.2	6.39 10.7	04/07/2014 27/07/2014	187.6 121.4	157.6124.5	4.5 4.9	9.82 6.66
18/04/2013 04/04/2013	98.4 128.5	111.3 124.5	1.9 4.2	7.80 10.7	30/09/2015 16/09/2015	131.1 109.4	118.5106.3	1.8 9.9	10.7 6.25
28/11/2013 15/11/2013	132.9 177.9	142.8 161.6	0.2 4.3	7.94 12.5	10/04/2016 27/04/2016	110.6 92.6	101.892.0	6.0 6.8	9.98 5.08
23/06/2014 11/06/2014	92.6 168.4	110.5 149.7	2.5 6.6	4.66 7.87	11/04/2016 27/04/2016	116.6 92.6	104.892.0	2.5 6.8	11.1 5.08
22/07/2014 27/07/2014	92.6 121.4	110.3 124.5	4.0 4.9	4.32 6.67	15/10/2016 21/10/2016	84.9 77.8	84.380.4	10.2 1.5	8.75 5.08
27/05/2015 08/05/2015	95.2 149.8	109.8 137.8	3.4 7.0	5.29 9.90	11/04/2017 12/04/2017	74.6 71.4	75.473.8	8.8 7.9	6.48 3.62
28/05/2015 08/05/2015	93.0 149.8	108.6 137.8	6.1 7.0	4.82 9.90	16/04/2017 12/04/2017	74.5 71.4	75.473.8	4.1 7.9	5.90 3.62
26/03/2016 02/03/2016	85.5 98.2	89.1 98.0	2.1 6.1	4.82 8.82	29/04/2017 12/04/2017	77.0 71.4	77.273.8	5.4 7.9	5.40 3.62

**Figure 1:**
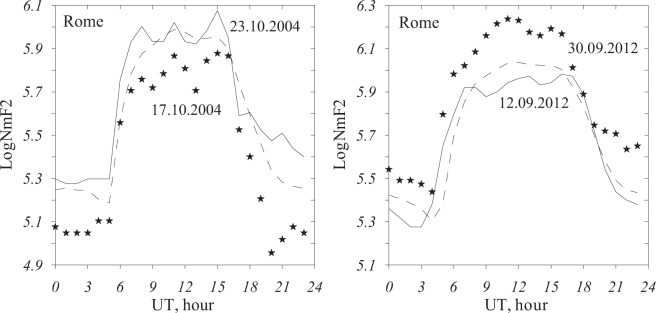
Diurnal variation of logN_m_F_2_ during negative (left panel) and positive (right panel). Q-disturbances at Rome. Asterisks – Q-disturbance variations, dashes – local model monthly medians, solid line – reference days close to the model monthly medians.

Besides the background level we need a particular day of the month which would be as much as possible close to the model background N_m_F_2_. Such days are needed to compare the retrieved aeronomic parameters to those retrieved for the Q-disturbance days. Examples of negative and positive Q-disturbances analyzed in the paper are given in Fig. 1.

If a day is marked as a Q-disturbed one then the sign of N_m_F_2_ deviations (negative or positive) is kept for many hours, sometimes for the whole day as in Fig. 1. This is different from usual geomagnetic storm induced F_2_-layer disturbances when positive and negative phases in N_m_F_2_ variations may change each other in the course of a day. Table 1 gives daytime Q-disturbances found at Rome for the period since 2002. Observed and averaged over (11–13) LT N_m_F_2_ values are given along with daily F_10.7_ and *ap(τ*) indices^30^

$${a}_{p}(\tau )=(1-\tau )\cdot \sum _{n=0}{a}_{p-n}{\tau }^{n},$$

where *a*_*p−n*_ (n = 0, 1, 2, 3…) – *ap* value for the current 3-hour interval, −3 h, −6 h, etc., τ = 0.75, *ap(τ*) values are given for 12 LT. This time-integrated index takes into account the prehistory of geomagnetic activity development manifesting more smooth variations and it is more appropriate for our analysis compared to usual 3 hour-*ap* indices.

Q-disturbances from Table 1 were developed with the method^13^ to retrieve a consistent set of the main aeronomic parameters responsible for the formation of the daytime mid-latitude F-layer. The method^13^ is based on solving an inverse problem of aeronomy using observed noontime f_o_F_2_ and five at (10,11,12,13,14) LT values of plasma frequency f_p180_ at 180 km height as input information along with standard indices of solar (F_10.7_) and geomagnetic (Ap) activity. Data on f_o_F_2_ and f_p180_ are available from DPS-4^14^ ground-based ionosonde observations after the ionogram reduction. The list of the retrieved parameters includes: neutral composition (O, O_2_, N_2_ concentrations), exospheric temperature Tex, the total solar EUV flux with λ ≤ 1050 Å, and vertical plasma drift W mainly related to the meridional thermospheric wind, Vnx. By fitting calculated N_m_F_2_ to observed ones the method provides h_m_F_2_ values which are useful for physical interpretation. For some of the selected cases in Table 1 it was possible to find neutral gas density observations with the Gravity field and steady state Ocean Circulation Explorer (GOCE https://earth.esa.int/web/guest/-/goce-data-access-7219), CHAllenging Minisatellite Payload (CHAMP ftp://anonymous@isdcftp.gfz-potsdam.de/champ/), and Swarm (https://earth.esa.int/web/guest/swarm/data-access) satellites. For these cases the observed neutral gas density (ρ) was incorporated into our method as a fitted parameter. Available neutral gas density observations in the daytime European sector were reduced to the location of ionosonde and 12 LT using the MSISE00 thermospheic model^31^ and the following expression:

$${\rho }_{station}={\rho }_{satellite}\frac{MSISE{00}_{station}}{MSISE{00}_{satellite}}$$

We have used three ρ observations from an orbit with the latitudes close to the latitude of ionosonde station and then (after the reduction to the ionosonde location and 12 LT) found the mean over three ρ values which were used in our analysis. Normally such three values are very close to each other. The retrieved with our method neutral gas density ρ = m_1_[O] + m_2_[O_2_] + m_3_[N_2_] does not include the contribution of [He] and [N]. Therefore the observed densities were corrected using MSISE00. Depending on conditions this correction may be up to (1–5)%. During this reduction the height of ρ observation was kept unchanged not to introduce an additional uncertainty related to unknown MSISE00 neutral temperature Tex for the particular days in question. The method using observed neutral gas densities was applied when ρ observations were available both for the Q-disturbed and the reference days.

## Results

### Negative Q-disturbances

Table 2 gives observed δN_m_F_2_ along with the observed total solar EUV (100–1200)Å flux^32^ (http://lasp.colorado.edu/lisird/) as well as inferred EUV flux, vertical plasma drift W, F_2_-layer maximum height h_m_F_2_, atomic oxygen concentration at 300 km in a comparison to MSISE00 model^31^ values, and exospheric temperature Tex. Table 2 indicates that selected Q-disturbances are rather strong: δN_m_F_2_ = N_m_F_2 Qday_/N_m_F_2 ref_ are (0.5–0.7) with the average value 0.63 ± 0.08. Negative Q-disturbance cases manifest lower F_10.7_ and as a rule lower *ap(τ*) indices compared to the reference days. The average F_10.7 Qday_/F_10.7ref_ = 0.79 ± 0.12 and the average *ap(τ*)_Qday_/*ap(τ*)_*ref*_ = 0.51 ± 0.36. Lower F_10.7_ and *ap(τ*) indices mean lower ionizing EUV flux and lower level of auroral activity.

**Table 2:** Observed δN_m_F_2_ = N_m_F_2 Qday_/N_m_F_2 ref_, observed EUV along with inferred EUV fluxes, vertical plasma drift W, h_m_F_2_, atomic oxygen concentration at 300 km in a comparison to MSISE00 model values, and exospheric temperature Tex for negative Q–disturbance and reference days. Numbers in brackets are Q–disturbance day/Reference day ratios. Available Swarm (10^−16^), GOCE (10^−14^) and CHAMP (10^−15^) neutral gas densities at the satellite height are given in the last column. Dashes - observations are absent.

Date	δN_m_F_2_	EUV_obs_ ×10^−3^ Wm^−2^	EUV×10^10^ ph cm^−2^s^−1^	W m s^−1^	h_m_F_2_ km	Tex K	[O]_300_×10^8^ cm^−3^	[O]_300_×10^8^ cm^−3^ (MSISE00)	ρ_obs_ g cm^−3^
13/06/2002 08/06/2002	0.59	5.64 6.15 (0.92)	8.04 8.68 (0.93)	−0.6 0.0	341 352	1338 1308	9.83 11.98 (0.82)	7.55 7.89 (0.96)	— —
17/10/2004 23/10/2004	0.72	4.13 5.02 (0.82)	7.78 9.28 (0.84)	−16.3 −19.8	229 234	868 919	4.41 5.53 (0.80)	5.77 6.43 (0.90)	— —
15/08/2005 28/08/2005	0.59	3.46 4.15 (0.83)	6.57 7.36 (0.89)	−13.2 −9.4	234 247	841 928	2.71 4.01 (0.68)	3.48 4.08 (0.85)	2.59×10^−15^ 4.43×10^−15^ (0.58)
14/02/2006 19/02/2006	0.70	3.36 3.50 (0.96)	5.43 5.39 (1.01)	−27.4 −19.4	211 220	742 740	2.97 3.72 (0.80)	3.14 3.58 (0.88)	— —
27/01/2010 11/01/2010	0.76	3.42 3.50 (0.98)	5.44 5.89 (0.92)	−23.4 −23.8	212 213	740 750	2.06 2.84 (0.72)	3.21 3.49 (0.92)	— —
06/02/2010 11/02/2010	0.73	3.53 3.83 (0.92)	5.85 6.09 (0.96)	−28.1 −24.9	212 218	753 791	2.69 3.78 (0.71)	3.29 3.91 (0.84)	— —
18/12/2011 05/12/2011	0.56	5.17 5.82 (0.89)	7.97 8.76 (0.91)	−27.0 −19.2	230 243	925 980	5.82 7.82 (0.74)	6.77 8.19 (0.83)	— —
14/01/2012 07/01/2012	0.70	5.25 5.32 (0.99)	8.19 8.40 (0.98)	−26.8 −26.6	229 234	908 948	4.50 6.74 (0.67)	6.09 6.68 (0.91)	— —
06/12/2012 15/12/2012	0.68	4.34 4.94 (0.88)	7.79 9.51 (0.82)	−24.4 −23.8	220 236	856 922	3.94 5.58 (0.71)	5.48 6.28 (0.87)	— —
07/12/2012 15/12/2012	0.60	4.39 4.94 (0.89)	7.77 9.51 (0.82)	−23.7 −23.8	218 236	858 922	3.60 5.58 (0.64)	5.44 6.28 (0.87)	— —
17/04/2013 04/04/2013	0.60	4.46 5.01 (0.89)	8.84 9.66 (0.91)	−9.9 −9.0	261 265	1029 1013	4.90 6.11 (0.80)	7.08 7.32 (0.97)	6.90×10^−14^ 8.26×10^−14^ (0.83)
18/04/2013 04/04/2013	0.73	4.32 5.01 (0.86)	8.32 9.66 (0.86)	−10.3 −9.0	255 265	988 1013	5.22 6.11 (0.85)	6.83 7.32 (0.93)	6.78×10^−14^ 8.26×10^−14^ (0.82)
28/11/2013 15/11/2013	0.63	5.08 6.13 (0.83)	10.03 11.32 (0.89)	−30.5 −21.7	232 250	981 1074	6.21 8.24 (0.75)	8.06 9.53 (0.84)	— —
23/06/2014 11/06/2014	0.59	4.37 5.69 (0.77)	7.90 11.26 (0.70)	−5.1 −6.7	257 288	951 1116	3.29 4.87 (0.67)	4.94 6.75 (0.73)	4.44×10^−16^ 10.3×10^−16^ (0.43)
22/07/2014 27/07/2014	0.65	4.34 5.01 (0.87)	7.90 9.83 (0.80)	−10.1 −9.6	251 255	995 1004	3.94 4.33 (0.91)	4.68 5.25 (0.89)	6.47×10^−16^ 7.21×10^−16^ (0.90)
27/05/2015 08/05/2015	0.53	4.56 5.32 (0.86)	8.19 10.25 (0.80)	−11.4 −6.5	249 292	992 1083	4.27 6.71 (0.64)	5.64 7.57 (0.74)	8.47×10^−16^ 15.9×10^−16^ (0.53)
28/05/2015 08/05/2015	0.49	4.50 5.32 (0.85)	7.86 10.25 (0.77)	−13.7 −6.5	239 292	1010 1083	4.23 6.71 (0.63)	5.75 7.57 (0.76)	8.90×10^−16^ 15.9×10^−16^ (0.56)
26/03/2016 02/03/2016	0.55	3.93 4.42 (0.89)	7.40 8.12 (0.91)	−9.9 −10.7	241 238	858 885	3.06 4.80 (0.64)	4.84 5.12 (0.94)	4.44×10^−16^ 7.54×10^−16^ (0.59)

Table 2 manifests a systematic decrease of atomic oxygen concentration on Q-disturbance days compared to reference days. This is valid not only on average but for particular Q-disturbance cases as well. The average [O]_Qday_/[O]_ref_ ratio = 0.73 ± 0.08 and the difference from 1.0 is absolutely significant according to Student t-criterion. Atomic oxygen is a crucial parameter for the daytime F_2_-layer^33^ as N_m_F_2_ ∼ [O]^4/3^. Table 2 also indicates that the inferred difference in [O]_300_ between Q-disturbance and the reference days is systematically larger than MSISE00 predicts.

Along with decreased [O] concentration on Q-disturbance days ionizing solar EUV flux is also decreased on average by ∼13% due to lower level of solar activity. This decrease is seen both in observed (http://lasp.colorado.edu/lisird/) and retrieved EUV fluxes. Table 2 indicates the average observed EUV_Qday_/EUV_ref_ ratio 0.88 ± 0.06 while the retrieved average ratio is 0.87 ± 0.08. This closeness of observed and retrieved EUV ratios tells us that the method^13^ provides reasonable results.

### Positive Q-disturbances

The results on positive Q-disturbances are given in Table 3. The selected disturbances are rather strong with δN_m_F_2_ up to 2.2 (the average value =1.66 ± 0.24). In contrast to the negative Q-disturbances F_10.7_ and *ap(τ*) indices on average are larger compared to the reference days: F_10.7 Qday_/F_10.7ref_ = 1.16 ± 0.20 and *ap(τ*)_Qday_/*ap(τ*)_*ref*_= 1.78 ± 1.61. This means larger ionizing EUV flux and stronger auroral heating for Q-disturbance days.

**Table 3:** Similar Table 2 but for positive Q-disturbances.

Date	δN_m_F_2_	EUV_obs_ ×10^-3^ Wm^−2^	EUV×10^10^ ph cm^−2^s^-1^	W m s^−1^	h_m_F_2_ km	Tex K	[O]_300_×10^8^ cm^−3^	[O]_300_×10^8^ cm^−3^ (MSISE00)	ρ_obs_ g cm^−3^
23/08/2002 08/08/2002	1.85	7.74 5.82 (1.33)	12.76 9.97 (1.28)	−7.5 −9.3	316 293	1280 1154	10.36 7.94 (1.30)	10.10 8.01 (1.26)	— —
17/06/2004 28/06/2004	1.42	4.32 4.19 (1.03)	7.60 7.71 (0.99)	−0.1 −9.8	301 264	1071 994	6.96 5.30 (1.31)	4.83 4.35 (1.11)	— —
26/10/2004 23/10/2004	1.55	4.93 5.02 (0.98)	9.43 9.12 (1.03)	−8.9 −14.3	252 246	938 927	6.11 4.84 (1.26)	6.93 6.43 (1.08)	— —
10/06/2007 06/06/2007	1.45	3.51 3.60 (0.97)	6.32 6.75 (0.94)	−6.7 −11.1	246 229	878 868	3.99 2.70 (1.48)	3.27 3.16 (1.03)	4.83×10^−15^ 3.37×10^−15^ (1.43)
22/10/2009 13/10/2009	1.22	3.20 3.16 (1.01)	6.06 5.93 (1.02)	−15.8 −20.0	227 219	780 753	4.36 3.56 (1.22)	4.02 3.60 (1.12)	— —
15/02/2010 11/02/2010	1.71	3.86 3.83 (1.01)	7.42 7.70 (0.96)	−14.3 −20.6	235 217	818 788	4.51 2.83 (1.59)	4.08 3.91 (1.04)	— —
20/10/2010 03/10/2010	1.48	3.86 3.64 (1.06)	7.07 6.85 (1.03)	−14.8 −16.2	226 222	792 803	4.45 3.57 (1.25)	4.61 4.16 (1.11)	— —
11/06/2012 27/06/2012	1.76	5.30 4.57 (1.16)	10.26 9.02 (1.13)	−6.6 −12.8	284 250	1089 1004	6.87 4.46 (1.54)	6.02 5.03 (1.20)	— —
30/09/2012 12/09/2012	1.99	5.61 4.85 (1.16)	10.49 8.61 (1.22)	−7.4 −8.6	278 256	1012 943	8.10 5.02 (1.61)	7.38 6.08 (1.21)	7.76×10^−14^ 5.33×10^−14^ (1.46)
25/08/2013 01/08/2013	1.59	5.21 4.97 (1.05)	9.19 9.24 (0.99)	−6.1 −10.0	267 250	974 961	5.81 4.06 (1.43)	5.30 4.63 (1.14)	— —
04/07/2014 27/07/2014	1.47	6.32 5.10 (1.24)	11.76 9.81 (1.20)	−9.1 −10.3	273 252	1111 997	5.77 4.42 (1.30)	6.17 5.25 (1.17)	12.53×10^−16^ 7.21×10^−16^ (1.74)
30/09/2015 16/09/2015	1.71	5.04 4.40 (1.14)	9.53 8.54 (1.12)	−11.5 −7.2	249 251	943 907	5.76 3.58 (1.61)	6.23 5.57 (1.12)	8.70×10^−16^ 5.95×10^−16^ (1.46)
10/04/2016 27/04/2016	1.96	4.04 4.10 (0.98)	8.51 7.88 (1.08)	−1.3 −18.8	287 229	1036 937	6.93 4.05 (1.71)	5.65 4.99 (1.13)	— —
11/04/2016 27/04/2016	2.19	3.98 4.10 (0.97)	8.62 7.88 (1.09)	−3.0 −18.8	275 229	994 937	6.62 4.05 (1.63)	5.50 4.99 (1.10)	— —
15/10/2016 21/10/2016	1.72	3.80 3.53 (1.08)	7.33 6.69 (1.09)	−10.3 −13.7	244 227	851 789	4.91 2.90 (1.69)	5.15 4.08 (1.26)	— —
11/04/2017 12/04/2017	1.79	3.04 3.07 (0.99)	6.48 6.08 (1.06)	−8.1 −10.0	247 235	856 831	4.57 2.81 (1.62)	4.33 4.23 (1.02)	— —
16/04/2017 12/04/2017	1.63	3.29 3.07 (1.07)	6.28 6.08 (1.03)	−10.2 −10.0	237 235	824 831	3.93 2.81 (1.40)	4.07 4.23 (0.96)	— —
29/04/2017 12/04/2017	1.49	3.47 3.07 (1.13)	6.51 6.08 (1.07)	−10.6 −10.0	236 235	842 831	3.51 2.81 (1.25)	4.15 4.23 (0.98)	— —

In contrast to negative Q-disturbances the concentration of atomic oxygen [O] is systematically larger on Q-disturbance days compared to reference ones. This is valid not only on average but for individual Q-disturbance cases as well. Table 3 shows that average [O]_Qday_/[O]_ref_ ratio is 1.46 ± 0.17, the difference from 1.0 being absolutely significant according to Student t-criterion. A comparison to the MSISE00 model (Table 3) shows that the model systematically underestimates the atomic oxygen variations. The same situation we met analyzing negative Q-disturbances.

Table 3 also demonstrates that Q-disturbance days on average are characterized by larger (∼7%) solar EUV fluxes and this increase is confirmed by direct EUV observations (http://lasp.colorado.edu/lisird/): the inferred average EUV_Qday_/EUV_ref_ ratio is 1.07 ± 0.09 while the observed ratio is 1.08 ± 0.10. By analogy with negative Q-disturbances (Table 2) the retrieved and observed average EUV_Qday_/EUV_ref_ ratios practically coincide and this indicates that the method^13^ provides reasonable results as the observed EUV fluxes have nothing common with the retrieval process.

Along with this Table 3 shows some cases with the EUV_Qday_/EUV_ref_ ratio ≤ 1.0. Electron concentration at F_2_-region heights depends on the O^+^ ion production rate q(O^+^) ∼ I_EUV_[O], where I_EUV_ – the intensity of incident solar EUV radiation. Table 4 gives positive disturbance cases with EUV_Qday_/EUV_ref_ <1.0 from Table 3. The ratio R = (EUV × [O]_300_)_Qday_/(EUV × [O]_300_)_ref_ is >1.0 for such cases as well. It should be noted that R is always <1.0 for negative Q-disturbances (Table 2). Therefore the I_EUV_[O] product is always >1.0 for positive Q-distubances and it is <1.0 for negative Q-disturbances. The same rule is valid for atomic oxygen concentration as this was shown earlier. Therefore both aeronomic parameters may be used to divide Q-disturbances into negative and positive ones. This result is important from practical point of view as it can be used to predict the type of Q-disturbance.

**Table 4:** (EUV×[O]_300_)_Qday_/(EUV×[O]_300_)_ref_ ratio for positive. Q-disturbance cases with EUV_Qday_/EUV_ref_ < 1.0.

Q day Ref day	17/06 28/06 2004	10/06 06/06 2007	15/02 11/02 2010	25/08 01/08 2013
Ratio	1.30	1.39	1.53	1.42

### Interpretation

The undertaken analysis has shown that strong (with magnitude >35%) daytime both negative and positive Q-disturbances in N_m_F_2_ are mainly due to day-to-day variations of atomic oxygen concentration. The average [O]_Qday_/[O]_ref_ ratio = 0.73 ± 0.08 for negative and 1.46 ± 0.17 for positive Q-disturbances.

In both cases the difference with respect to 1.0 is absolutely significant according to Student t-criterion. This day-to-day difference takes place not only on average but for individual Q-disturbance cases as well.

This is a new and principle result. Partly [O] variations may be attributed to day-to-day changes in solar (F_10.7_ index) and geomagnetic (Ap index) activity (Table 1). However MSISE00 driven by these indices systematically underestimates the required (retrieved) day-to-day [O] variations (Tables 2 and 3).

Next in the hierarchy of contributors is standing solar EUV. Both observed and retrieved total solar EUV fluxes indicate on average a decrease for negative EUV_Qday_/EUV_ref_ = 0.88 ± 0.06 (the retrieved 0.87 ± 0.08) and an increase for positive EUV_Qday_/EUV_ref_ = 1.08 ± 0.10 (the retrieved 1.07 ± 0.09) Q-disturbances. Note the closeness between the observed and retrieved EUV_Qday_/EUV_ref_ average ratios.

Of course, EUV reflects the corresponding variations of solar activity. The EUVAC^34^ empirical model is based on the F = (F_10.7_ + F81)/2 index of solar activity where F81 is 81- day average of daily F_10.7_ centered on the day in question (Table 1). Average of the F_Qday_/F_ref_ ratio = 0.89 ± 0.07 for negative and 1.08 ± 0.09 for positive Q-disturbances. This is very close to the observed average EUV_Qday_/EUV_ref_ ratios.

Some but not large contribution to N_m_F_2 Qday_/N_m_F_2 ref_ difference provides vertical (downward) plasma drift W presumably related to meridional thermospheric wind Vnx = W/sinIcosI, where I- magnetic inclination at a given location. We are speaking about an effective meridional thermospheric wind. Average W = −17.3 ± 9.1 m/s for negative Q-disturbance days and W = −15.0 ± 8.2 m/s for reference days (Table 2) while average W = −8.5± 4.3 m/s for positive Q-disturbance days and W = −12.9 ± 4.3 m/s for reference days (Table 3). According to theory of ionospheric F_2_-layer the stronger downward plasma drift the less N_m_F_2_ i.e. stronger poleward Vnx increases the negative Q-disturbance effect while damped poleward Vnx increases the positive Q-disturbance effect.

Negative Q-disturbance days are characterized by lower h_m_F_2_ while positive Q-disturbance days – by larger h_m_F_2_ compared to reference days. Tables 2 and 3 give the average (h_m_F_2 Qday_ − h_m_F_2 ref_) difference of −14. 3 km for negative Q-disturbance cases and this difference is +18.9 km for positive Q-disturbances.

The following expression for h_m_F_2_ obtained from a solution of the continuity equation for electron concentration in the stationary daytime mid-latitude F_2_-layer^35^ gives the h_m_F_2_ dependence on aeronomic parameters

1 $${h}_{m}{F}_{2}=\frac{H}{3}[\mathrm{ln}({\beta }_{1}\cdot {[O]}_{1})+\,\mathrm{ln}({H}^{2}/(0.54d))]\,+cW+{h}_{1}$$

where *H* = *kT/mg* – scale height and [*O*]_1_ concentration of neutral atomic oxygen at a fixed height h_1_ (say 300 km), *β* = *γ*_1_[N_2_] + *γ*_2_[O_2_] – linear loss coefficient, *d* = *D* × [*O*]_1_*, D* – ambipolar diffusion coefficient at *h*_1_ height, *W* – vertical plasma drift, *c* – a constant. Expression (1) indicates a linear h_m_F_2_ dependence on neutral temperature and vertical plasma drift while the dependence on neutral composition is weaker via logarithm. Larger average Tex and W on positive Q-disturbance days compared to reference ones provide larger h_m_F_2_ (Table 3), the inverse situation takes place for negative Q-disturbance days (Table 2).

Earlier it was stressed that atomic oxygen day-to-day variations determined the type (positive or negative) of N_m_F_2_ Q-disturbances. Therefore a mechanism of [O] day-to-day variations is a principle question. It is well-known that atomic oxygen is totally produced and lost in the upper atmosphere^36^. It is produced via O_2_ photo-dissociation above ∼120 km then molecular diffusion transfers it downward to the turbopause (100–110) km level and further it is transferred downward by eddy diffusion. The maximum in the atomic oxygen height distribution around 97 km is formed in the downward eddy diffusion flux with the exponentially increasing association of [O] via a three-body collision O + O + M → O_2_ + M^37,38^, where M is a total number density of neutral species mostly presented by [N_2_]. Along with these local processes atomic oxygen is transferred by global thermospheric circulation and this process is very efficient.

Day-to-day variations of solar dissociative radiation in Schumann-Runge continuum in principle may contribute to variations of the atomic oxygen abundance when Q-disturbance and the reference days are largely separated in time as the characteristic (e-fold) time of the O_2_ dissociation process is ∼ 1/J_O2_ ≤ 3 days above 120 km height^36^ (their Fig. 8.2). However sometimes Q-disturbance and reference days are neighboring ones (e.g. 11/04/2017 and 12/04/2017, Table 3) and this supposes a very fast redistribution of atomic oxygen.

The annual mean eddy diffusion coefficient is ∼4 × 10^6^ cm^2^/s at 85–100 km^39^. This gives the characteristic time for the eddy diffusion transfer of atomic oxygen τ_tr_ ∼ H^2^/K_edd_ ∼ 1 day.

This time is comparable to the observed times of atomic oxygen changes during Q-disturbance events.

However at present it is not known how eddy diffusion is related to day-to-day changes in solar and geomagnetic activity clearly seen in Q-disturbances occurrence. Moreover K_edd_ manifests seasonal variations^40^ which are not followed by Q-disturbance occurrences.

Therefore global thermospheric circulation looks like the most preferable process to explain day-to-day variations of the atomic oxygen abundance. Negative Q-disturbances are associated with extremely low level of magnetic activity corresponding to the minimal intensity of auroral heating. This corresponds to an unconstrained solar-driven thermospheric circulation (a strong poleward neutral Vnx wind during daytime hours) and to relatively low atomic oxygen concentrations at middle latitudes as this follows from the model simulations^41^ - low [O] may be related to a moderate upwelling of neutral gas in a wide range of latitudes (their Fig. 3). Indeed, retrieved W for negative Q-disturbance days on average are larger compared to reference days (see earlier) and average [O]_Qday_/[O]_ref_ ratio = 0.73 ± 0.08. The available neutral gas density observations confirm the obtained results. On negative Q-disturbance days the observed neutral gas density at the satellite height is systematically lower compared to the reference days (Table 2). This difference in neutral gas density is due to different Tex and atomic oxygen concentration.

Similar explanations may be applied to positive Q-disturbance cases. Smaller vertical plasma drifts W on Q-disturbed days (Table 3) tell us that the northward circulation was damped. This should decrease or even invert upwelling increasing by this way the atomic oxygen abundance in the thermosphere^41,42^.

Indeed, the retrieved average W = −8.5 ± 4.3 m/s for positive Q-disturbance days while W = −12.9 ± 4.3 m/s for reference days, along with this the average [O]_Qday_/[O]_ref_ ratio is 1.46 ± 0.17 (Table 3).

Direct observations of neutral gas density for Q-disturbance and reference days confirm the obtained results. Four events with ρ observations (Table 3) indicate an increase of neutral gas density on Q-disturbance days. This increase is due to larger neutral temperature and larger atomic oxygen concentration at the analyzed heights >400 km. The main contribution to ρ variations provides atomic oxygen as this follows from a comparison of 10/06/2007 and 06/06/2007 (Table 3). The difference in Tex was not large (∼10 K) for these days while [O]_Qday_/[O]_ref_ = 1.48 and ρ_Qday_/ρ_ref_ = 1.43 are very close.

It would be interesting to follow the quantitative contribution of different aeronomic parameters to the observed negative and positive Q-disturbances. This can be done using an approximate expression^43^ for N_m_F_2_ in the day-time mid-latitude ionosphere:

2 $${N}_{m}{F}_{2}=0.75\frac{{q}_{m}}{{\beta }_{m}}$$

where q_m_ – O^+^ ion production rate and β_m_ – linear loss coefficient taken at the height of the F_2_-layer maximum, h_m_F_2_. This expression does not take into account the contribution of vertical plasma drift W. For this reason we should choose such cases where drifts were not large and close for the Q-disturbance and reference days to minimize possible effects of the W neglect. Two cases from Tables 2 and 3 (13/06/2002)/(08/06/2002) and (23/08/2002)/(08/08/2002) can be used for such analysis. Table 5gives necessary aeronomic parameters taken from our calculations.

**Table 5:** Inferred aeronomic parameters at h_m_F_2_ for negative (top lines) and positive (bottom lines). Q-disturbances. The last columns give inferred δ(q/β)_max_ and observed δN_m_F_2._.

Date	h_m_F_2_ km	Tex, K	[O]_max_ 10^8^,cm^−3^	[N_2_]_max_ 10^8^,cm^−3^	(O/N_2_)_max_	q_max_×10^2^ cm^−3^ s^−1^	β_max_×10^−4^, s^−1^	δ(q/β)_max_	δN_m_F_2_,_obs_
13/06/2002	341	1338	5.69	1.75	3.24	2.36	2.11	0.58	0.59
08/06/2002	352	1308	5.96	1.19	5.02	2.80	1.45		
23/08/2002	316	1280	8.26	1.90	4.34	5.52	2.13	1.84	1.85
08/08/2002	293	1154	8.82	3.06	2.88	4.66	3.30		

The approximate expression (2) is seen to describe satisfactorily the observed δN_m_F_2_ both for the selected negative and positive Q-disturbances (Table 5). The negative Q-disturbance is due to lower q_max_ and larger β_max_ compared to the reference day while this relation is inversed for the positive Q-disturbance. To a first approximation q_max_ ∼ I_EUV_ × [O]_max_ and β_max_ ∼ [N_2_]_max_ therefore N_m_F_2_ ∼ [O]_max_/[N_2_]_max_ and Table 5 confirms this (see (O/N_2_)_max_ ratio): 3.24/5.02 = 0.64 (observed 0.59) in the case of negative Q-disturbance and 4.34/2.88 = 1.51 (observed 1.85) for the positive Q-disturbance. The residual difference may be attributed to the difference in EUV. The EUV flux is less on the negative Q-disturbance day and it is larger on the positive Q-disturbance day compared to the reference day (Tables 2 and 3).

## Discussion

At the beginning of our investigations we supposed that F_2_-layer Q-disturbances could be related to the impact from below – the so-called “meteorological control” of the Earth’s ionosphere^1–6^. Seismic events seem also to affect the F_2_-region^44,45^. We ourselves have also considered ionospheric precursors for crustal earthquakes in Italy^46^. However all these morphological and correlation analyses are still at the level of pure speculations concerning physical mechanisms of such impact on the ionosphere. Observed N_m_F_2_ variations with duration longer than e-fold time (∼1.5 hour) imply changes in production and recombination rates i.e. in neutral composition and temperature or changes in thermospheric winds or electric fields. Therefore, telling about the impact from below firstly it is necessary to show which aeronomic parameters responsible for the F_2_-layer formation have been changed and secondly what is the mechanism of such changes, if it is plausible from physical point of view. Unfortunately up to now we don’t have necessary observations to answer the formulated questions.

With our recently proposed method^13^ the first step in this direction has been done. For a particular geophysical situation using the observed changes of electron concentration in F_1_ and F_2_ layers the method tells us how the main (controlling) aeronomic parameters have been changed. The method is confined to middle latitudes and noontime hours. However it is sufficient to analyze mid-latitude daytime long-lasting (≥3 hours) Q-disturbances taking place under magnetically quiet conditions.

Using Rome ionosonde observations about forty of analyzed Q-disturbance cases have shown the crucial role of atomic oxygen day-to-day variations in producing F_2_-layer Q-disturbance both negative and positive ones. It was shown that atomic oxygen was systematically decreased on negative Q-disturbance days (Table 2) and it is systematically increased on positive Q-disturbance days (Table 3) compared to reference days. This result may be considered as a principle one – it was obtained directly from ionospheric observations for the dates of Q-disturbance events. It should be stressed that a good empirical thermospheric model MSISE00^31^ does not reproduce (for understandable reasons) the required day-to-day variations of atomic oxygen (Tables 2 and 3).

CHAMP, GOCE, and Swarm satellite neutral gas density (ρ) observations were used as a direct confirmation to the obtained variations of atomic oxygen on Q-disturbance days. Neutral gas density at satellite heights (>400 km) is mainly presented by atomic oxygen. Therefore the retrieved [O] variations should be seen in satellite ρ observations. Indeed, Tables 2 and 3 show that observed neutral gas densities are systematically lower for negative and larger for positive Q-disturbance days compared to reference days. Moreover when Tex are close for the Q-disturbed and reference days neutral gas density variations should be close to corresponding variations of atomic oxygen concentration. Two examples on 22/07/2014 for negative Q-disturbance and on 10/06/2007 for positive one confirm this: the inferred [O]_Qday_/[O]_ref_ ratios practically coincide with the observed ρ_Qday_/ρ_ref_ ratios (Tables 2 and 3). All this tells us about the reality of the obtained result on the leading role of atomic oxygen in the formation of Q-disturbances.

**Figure 2:**
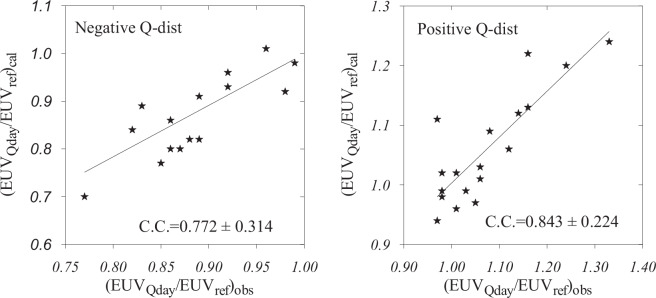
Retrieved EUV_Qday_/EUV_ref_ versus observed ratios for negative and positive Q-disturbance cases. Correlation coefficients (C.C.) along with the confidence intervals are shown.

Some contribution in a proper direction provides solar ionizing EUV radiation. Both observed and retrieved total solar EUV fluxes indicate on average a decrease for negative EUV_Qday_/EUV_ref_ = 0.88 ± 0.06 (the retrieved 0.87 ± 0.08) and an increase for positive EUV_Qday_/EUV_ref_ = 1.08 ± 0.10 (the retrieved 1.07 ± 0.09) Q-disturbances. It should be noted the closeness between the observed and retrieved EUV_Qday_/EUV_ref_ average ratios. These average results were obtained from individual comparisons given in Fig. 2.

The correlation coefficients between observed and retrieved EUV_Qday_/EUV_ref_ ratios are significant at the 99.9% confidence level according to Student criterion. This comparison may be considered as an absolutely independent control of the applied method^13^ as the observed EUV (http://lasp.colorado.edu/lisird/) fluxes have nothing common with our method to retrieve aeronomic parameters from ionospheric observations.

The crucial role of atomic oxygen in the formation of daytime F_2_-layer Q-disturbances has been shown by the undertaken analysis. The other and more difficult question – what is the cause of such day-to-day variations of atomic oxygen? Our earlier discussion of this issue has shown that global thermospheric circulation which is resulted from a competition between solar-driven (background) and storm-induced (due to auroral heating) circulations looks as a plausible mechanism. The inferred W and h_m_F_2_ variations (Tables 2 and 3) clearly indicate changes in the effective meridional wind Vnx when we pass from a reference day to a Q-disturbance one. Under low geomagnetic and lower solar activity compared to the background level we have an unconstrained solar-driven circulation with a strong northward Vnx decreasing the atomic oxygen abundance at F_2_-layer heights^41,42^. These factors along with lower EUV flux work in one direction to decrease N_m_F_2_ and create a negative Q-disturbance. Under slightly elevated geomagnetic and larger solar activity compared to the background level we have damped northward solar-driven circulation. This takes place for two reasons: an elevated auroral heating and an increased ion drag due to larger electron concentration. Damped poleward circulation increases the atomic oxygen abundance in the mid-latitude thermosphere due to downwelling^41^. This along with larger EUV flux creates conditions for a positive Q-disturbance to occur. It should be stressed that all this takes place under quiet geomagnetic conditions when daily Ap <15 nT. Under larger geomagnetic activity (Ap > 15–20 nT) auroral heating increases and inverts the solar-driven thermospheric circulation. The disturbed neutral composition moved by the disturbed equatorward wind spreads from the auroral zone to middle latitudes and we obtain a normal F_2_-layer storm-induced disturbance. Therefore in the case of Q-disturbances we have the same F_2_-layer storm mechanism^41,42,47–49^ manifesting itself under low geomagnetic activity.

In this paper we have analyzed the simplest situation with strong Q-disturbances at middle latitudes during daytime hours. According to our morphological analysis^9^ such events are not numerous while the majority of Q-disturbances take place during evening and nighttime hours. The nighttime and evening F_2_-layer formation mechanisms are strongly related and meridional thermospheric wind plays an essential role in this mechanism. Future considerations of Q-disturbances in different LT sectors may help clear up the role of thermospheric circulation in day-to-day atomic oxygen variations.

## Conclusions

For the first time negative and positive near noontime prolonged (≥3 hours) F_2_-layer Q-disturbances (daily Ap <15 nT) observed with a ground-based ionosonde at Rome under various seasons and levels of solar activity have been analyzed to reveal their formation mechanism. The recently developed method^13^ to extract a consistent set of the main aeronomic parameters from f_p180_ and f_o_F_2_ observations has been used for this analysis. The obtained results may be summarized as follows.

1. Day-to-day atomic oxygen variations at F_2_-region heights specify the type (positive or negative) of N_m_F_2_ Q-disturbances with the average [O]_Qday_/[O]_ref_ ratio at 300 km 1.46 ± 0.17 for positive Q-disturbance and 0.73 ± 0.08 for negative ones. This difference in [O] between Q-disturbance and reference days takes place not only on average but for all individual cases in question. The required atomic oxygen day-to-day variations are not described by the empirical MSISE00 thermospheric model which systematically underestimates the magnitude of these variations.
2. The retrieved atomic oxygen day-to-day variations are confirmed by CHAMP, GOCE, and Swarm satellite neutral gas density observations. Neutral gas density at satellite heights (>400 km) which is mainly presented by atomic oxygen also manifests positive and negative deviations in accordance with the observed type of Q-disturbance.
3. An additional contribution to Q-disturbances formation is provided by solar EUV day-to-day variations. Both observed and retrieved total solar EUV fluxes indicate on average a decrease for negative EUV_Qday_/EUV_ref_ = 0.88 ± 0.06 (the retrieved 0.87 ± 0.08) and an increase for positive EUV_Qday_/EUV_ref_ = 1.076 ± 0.10 (the retrieved 1.074 ± 0.09) Q-disturbances. The closeness between observed and retrieved average EUV_Qday_/EUV_ref_ ratios is a direct confirmation for the reality of the obtained results as the observed EUV fluxes have nothing common with the retrieval process.
4. Negative Q-disturbance days are characterized by lower h_m_F_2_ (on average −14.3 km) while positive Q-disturbance days – by larger h_m_F_2_ (on average +18.9 km) compared to reference days. This is due to larger average Tex and vertical plasma drift W on positive Q-disturbance days compared to reference ones, the inverse situation takes place for negative Q-disturbance days.
5. Day-to-day changes in global thermospheric circulation manifested by W day-to-day variations are considered as a plausible mechanism. The analyzed type of F_2_-layer Q-disturbances can be explained in the framework of contemporary understanding of the thermosphere-ionosphere interaction based on solar and geomagnetic activity as the main drivers.
